# Diabetic Muscle Infarction

**DOI:** 10.5811/cpcem.2019.10.45483

**Published:** 2020-01-21

**Authors:** Sophia Ahmed, Romeo Fairley

**Affiliations:** UT Health San Antonio, Department of Emergency Medicine, San Antonio, Texas

## Abstract

A 58-year-old male with past medical history of diabetes mellitus presented with pain to the bilateral groin for six weeks. Magnetic resonance imaging of the patient’s lower extremities revealed acute myoedema, and he was diagnosed with myositis secondary to diabetic muscle infarction.

## CASE PRESENTATION

A 58-year-old male presented after persistent severe pain to the bilateral groin for six weeks. He had no fever, urinary symptoms, incontinence, trauma, weight loss, or saddle anesthesia. He had a history of diabetes mellitus and hypertension. Physical examination showed tenderness to palpation of the suprapubic region, normal testicles, no inguinal hernias, and hyperpigmentation extending from the groin to the left mid-thigh. Laboratory tests revealed an elevated creatinine of 1.56 milligrams per deciliter (mg/dL) (normal 0.60 to 1.30 mg/dL), creatine kinase (CK) of 177 units per liter (U/L) (normal 24 – 223 U/L), C-reactive protein (CRP) of 180 mg/L (normal <10.01 mg/L) and erythrocyte sedimentation rate (ESR) 106 millimeters per hour (mm/hr) (normal 2 – 37 mm/hr). Hemoglobin A1c was 11.4% (normal 4.0 – 6.4%), consistent with his longstanding history of poorly controlled diabetes.

Ultrasound of scrotum and computed tomography of the abdomen/pelvis were both non-diagnostic. Magnetic resonance imaging (MRI) of the bilateral lower extremities (see image), revealed acute myoedema involving the left obturator internus, externus and proximal adductor muscles of the left thigh. Findings were concerning for acute myositis likely secondary to diabetic muscle infarction. The patient was admitted for pain and glucose control. CRP down-trended to 110 mg/L prior to discharge and acute kidney injury resolved.

## DISCUSSION

Diabetic muscle infarction is a rare, difficult-to-diagnose disease causing significant pain and morbidity for patients. The mean duration of symptoms before presenting for care is about four weeks, and time to resolution ranges from 2–17 weeks with an average of four weeks.^1^ CK, ESR and CRP may be elevated, but are nonspecific. MRI with intravenous contrast is the most useful diagnostic imaging technique.^2^ Computed tomography and ultrasounds are routinely non-diagnostic. All patients with diabetic muscle infarction should be treated with an antiplatelet agent, most commonly aspirin.^3^ Use of antiplatelet and/or anti-inflammatory agents decreases mean recovery time by 2.5 weeks.^4^ Recurrence rates exceed 40%, so early recognition and management is pivotal.^4^

**CPC-EM Capsule**

What do we already know about this clinical entity?

The pathogenesis of myositis is unknown, and it is rarely diagnosed. It causes acute or subacute swelling, pain and tenderness, predominantly in the thigh or calf.

What is the major impact of the image(s)?

Magnetic resonance imaging demonstrates myositis in a typical distribution for diabetic muscle infarction. It may be undetected on other imaging modalities including computed tomography.

How might this improve emergency medicine practice?

Diabetes has increased in prevalence, so physicians should keep the diagnosis in mind. Magnetic resonance imaging is the diagnostic tool of choice.

## Figures and Tables

**Image:**
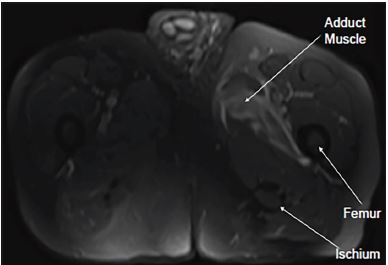
Magnetic resonance imaging demonstrating acute myositis of the left proximal adductor muscles.
